# BAG-3 Mutation Dilated Cardiomyopathy With Left Ventricular Noncompaction in Young Healthy Adult

**DOI:** 10.1016/j.jaccas.2024.102648

**Published:** 2024-11-20

**Authors:** Yash B. Patel, Wadie David, Milan Terzic, Akanksha Mehla, Tanmay V. Swadia

**Affiliations:** a Department of Internal Medicine, Trinity Health Ann Arbor, Michigan, USA; b Department of Cardiology, Ascension Southfield, Michigan, USA; c Department of Cardiology, University of Michigan Health Sparrow Hospital, Michigan, USA; d Trinity Health Michigan Heart, Michigan, USA

**Keywords:** cardiomyopathy, genetics, genetic disorders

## Abstract

Cardiomyopathies are generally divided into ischemic and nonischemic types. Dilated cardiomyopathies, which are a type of nonischemic cardiomyopathy, may have a trait of left ventricular noncompaction. We present the case of a 34-year-old man with new-onset decompensated heart failure and left ventricular noncompaction from a BAG3 (Bcl-2 associated athanogene 3) truncating mutation.

## Graphical Abstract

**Figure:**
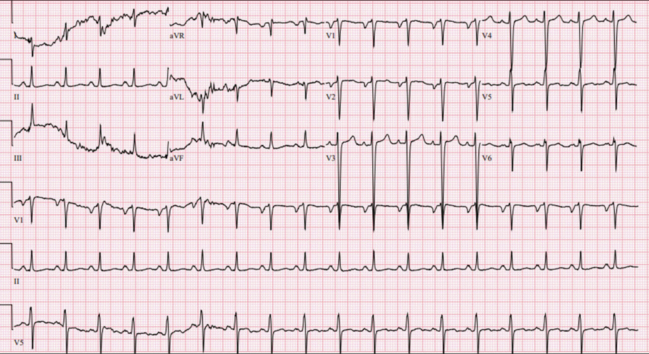

Cardiomyopathies encompass a diverse group of heart muscle diseases, varying in presentation. Traditionally, they have been categorized into ischemic and nonischemic types, with ischemic being more common. Among nonischemic cardiomyopathies, >30 genes have been identified with variants associated with the development of dilated cardiomyopathy (DCM).^1^ Identifying specific genetic mutations associated with DCM may play a crucial role in predicting the risk of disease development and targeted disease-specific treatment.^2^^,^^3^

**Take-Home Message**

• Early identification of rare forms of DCM and genetic mutations will help to enrich knowledge for physicians treating patients with such conditions and establishing treatment guidelines.

Left ventricular noncompaction (LVNC) is a trait that may be seen with DCM. This phenotype is believed to be associated with the BAG3 (Bcl-2 associated athanogene 3) mutations. We present a case of a 34-year-old Latin American male immigrant admitted with new-onset decompensated heart failure found to have DCM with LVNC. Genetic testing revealed a BAG3 truncating mutation at Trp26Ter.

## History of Presentation

A 34-year-old man of Argentinean heritage presented to the emergency department reporting a cough and difficulty breathing worsening for approximately 2 weeks. His initial vital signs were as follows: heart rate of 100 beats/min, blood pressure of 120/86 mm Hg, and respiratory rate of 18 breaths/min. Physical examination included signs of severe fluid overload, including bilateral lower extremity edema, elevated jugular venous distention, bilateral crackles, and a distended abdomen.

## Past Medical History

The patient has no previous medical history.

## Differential Diagnosis

The differential diagnosis includes myocarditis (possible viral or idiopathic) and cardiomyopathy, ischemic or nonischemic (infectious, substance use related, stress induced, or genetic).

## Investigations

Results of the patient’s initial laboratory tests were notable for elevated brain natriuretic peptide levels at 1,266 pg/mL and high-sensitivity troponin levels at 209 ng/L, peaking at 212 ng/L.

**Figure 1:**
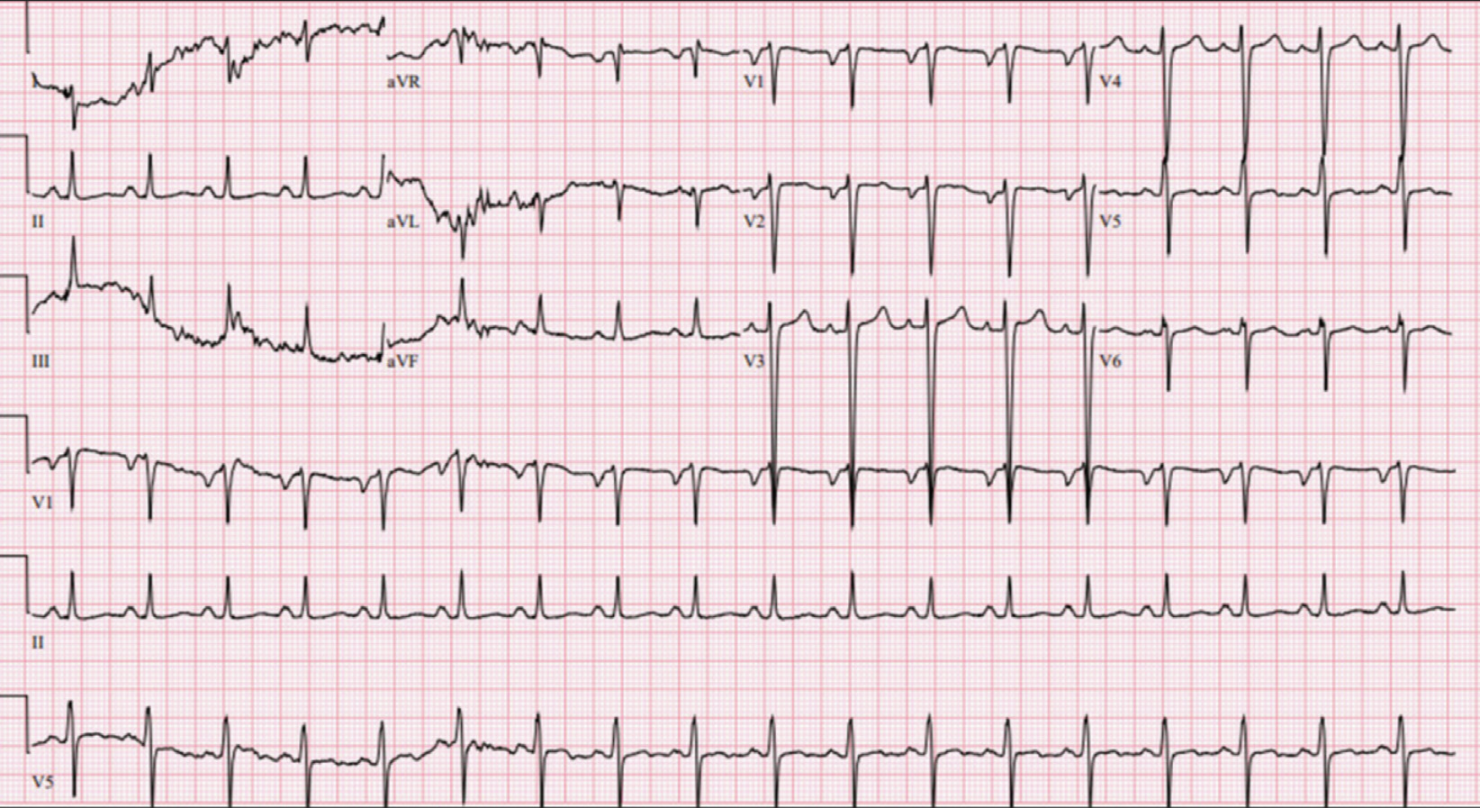
Electrocardiogram Showing Sinus Tachycardia

An electrocardiogram revealed sinus tachycardia (heart rate 116 beats/min) and no acute ischemic changes (Figure 1). A transthoracic echocardiogram revealed severe left ventricular (LV) global hypokinesis and a severely dilated left ventricle with an LV ejection fraction (LVEF) of 13%. The patient was also found to have a large 3.9 × 1.9 cm thrombus attached to the anterolateral wall and an additional 1.9 cm × 1.9 cm thrombus attached to the apex.

Results of the infectious work-up, which included *Trypanosoma* infection, Coxsackievirus B antibodies, human herpesvirus-6 antibodies, adenovirus antibodies, Epstein-Barr virus, and HIV, were all negative. Autoimmune etiologies were considered; test results for antinuclear antibodies, anti-neutrophil cytoplasmic antibodies, myeloperoxidase antibodies, proteinase 3 antibodies, anti-mitochondrial antibody, and anti-smooth muscle antibodies were negative.

**Figure 2:**
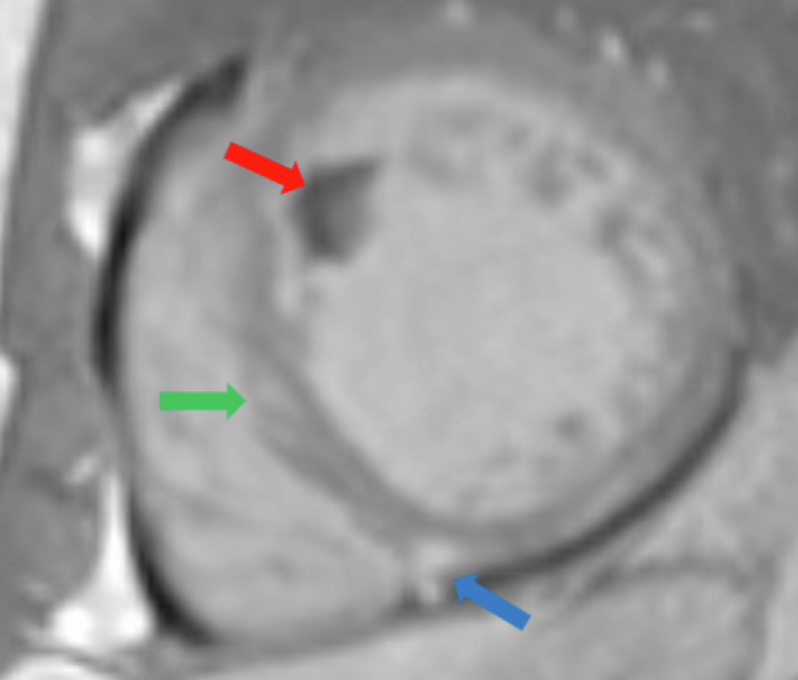
Thrombus on Cardiac MRI Apical thrombus (red arrow), with focal gadolinium uptake at the right ventricular insertion point (blue arrow), which suggests a dilated cardiomyopathy with some septal uptake (green arrow) noted on short-axis delayed phase-sensitive inversion recovery.

**Figure 3:**
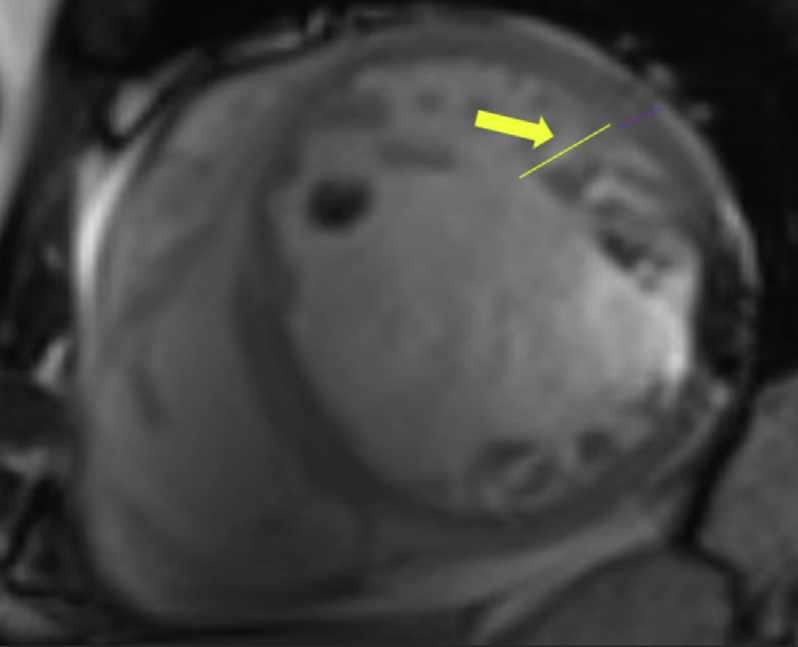
LV Apical Trabeculations Prominent apical trabeculations showing noncompacted myocardium best noted on end-diastole on cine short-axis (yellow arrow). The yellow line indicates the thickness of the noncompacted myocardium compared with compacted myocardium (purple).

Cardiac magnetic resonance imaging revealed late gadolinium enhancement, suggestive of nonischemic myocardial scarring or fibrosis in the basal and mid septum. Also noted were a 1.9 cm × 1.0 cm apical thrombus (Figure 2) and prominent LV apical trabeculations, suggestive of a noncompacted to compacted myocardium ratio of approximately 3.3 (>2.3 indicates LVNC) (Figure 3). There was no evidence of concomitant active inflammation.

## Management

Cardiac catheterization revealed normal coronary arteries.

The patient was initiated on therapy with apixaban 10 mg twice daily for a week followed by 5 mg twice daily for management of the thrombus.

The patient’s hospital course was complicated by cardiogenic shock, requiring dobutamine support. He was eventually initiated on guideline-directed medical therapy for heart failure with a discharge regimen of losartan 25 mg, spironolactone 12.5 mg, dapagliflozin 10 mg, and bisoprolol 2.5 mg. The patient was discharged with a wearable cardioverter defibrillator after 7 days in the hospital and referred to a heart transplant center.

## Outcome and Follow-Up

As an outpatient, the patient was enrolled in the CardioSeq registry roughly 2 months after discharge, where his genetic testing revealed a BAG3 point mutation at Trp26Ter. A transthoracic echocardiogram 6 months’ postdischarge revealed a persistently dilated left ventricle with improved systolic function of 28% as well as resolution of previously noted thrombus. The patient’s father with a known history of heart failure has not yet been tested for the BAG3 variant although he has been referred to a cardiologist.

## Discussion

BAG3 is a newly discovered DCM-associated gene accounting for approximately 2% of patients with DCM.^4^ BAG3 is essential for autophagy and maintenance of protein homeostasis.^5^ The gene encodes a 535 amino acid protein, and alterations have been shown to affect the myocardium, playing a role in the progression of DCM.^6^ In fact, in patients with DCM, between 2.3% and 15% of randomly selected probands involved BAG3 variations.^7^ Several disease-associated mutations in BAG3 have been documented, including 429 coding variants.^5^ Among these coding variants are 11 deletions, 33 nonsense (truncating) variants, 33 frame-shift (truncating) variants, and 351 missense (nontruncating) variants.^5^ Of the 33 truncating variants, 31 (93.94%) are categorized as pathogenic or likely pathogenic. Some nonsense truncating variants include p.Trp26Ter and p.Arg123Ter. The frequency of both mutations was 0.0008991% (1 of 111,226) and 0.001652% (2 of 121,084), respectively, suggesting the rarity of this mutation.^8^ One study found that truncating BAG3 mutations were associated with later disease onset compared with missense variants. At the age of 50 years, the occurrence of DCM was 90% among individuals with non-truncating BAG3 mutations compared with 55% among those with truncating mutations.^9^

A study of 129 individuals with BAG3 mutations causing DCM reported that the average age of diagnosis was 36.9 years and patients were predominantly NYHA functional class I at the time of initial diagnosis.^5^ One study found that in patients with abnormal diastolic patterns identified by tissue Doppler, this approach could be used to identify early-stage disease even with normal ejection fractions.^8^ Male sex was identified as a predictor of poorer outcomes compared with other causes of genetic DCM.^10^

Individuals with DCM resulting from a BAG3 mutation face an elevated risk of adverse cardiac events, with an annual incidence rate of 5.1%.^5^^,^^10^ These patients were also found to be treatment resistant, as only 2.9% had normalized LVEF during follow-up after the initial evaluation. Overall, clear guidelines are currently lacking for the phenotypic trait of LVNC, and whether there is optimal medical therapy for variants, reflected by minimal improvement in LVEF in the literature, is unknown.

## Conclusions

BAG3 mutation DCM with LVNC is aggressive and treatment resistant. It remains unknown if standard guideline-directed medical therapy is appropriate for these patients or if certain treatment regimens would be more optimal.

## Funding and Author Disclosures

The authors have reported that they have no relationships relevant to the contents of this paper to disclose.
